# Dental markers of biocultural sex differences in an early modern population from Gothenburg, Sweden: caries and other oral pathologies

**DOI:** 10.1186/s12903-021-01667-0

**Published:** 2021-06-14

**Authors:** Carolina Bertilsson, Lisa Nylund, Maria Vretemark, Peter Lingström

**Affiliations:** 1 Department of Cariology, Institute of Odontology, Sahlgrenska Academy, University of Gothenburg, Gothenburg, Sweden; 2 Västergötlands Museum, Skara, Sweden

**Keywords:** Dental anthropology, Caries, Diet, Sex-differences, Sixteenth century

## Abstract

**Background:**

With the aim to study dental pathological lesions in an early Swedish modern population, with special reference to sex variances of dental caries, the prevalence and distribution of dental caries and tooth wear were determined in complete and partial human dentitions from an early modern-time city graveyard (1500–1620) excavated in Gamlestaden, Gothenburg, Sweden.

**Methods:**

Partial and complete dentitions were examined through visual inspection and using a dental probe. Pathologies were studied, evaluated and presented by teeth and alveoli.

**Results:**

The study population consisted of 308 individuals. A total of 4,951 teeth in adults and 1,660 teeth in children were examined. Caries prevalence in the studied population was 55% and the highest prevalence of caries was found among the adults, where 68% of the individuals had at least one carious lesion. Caries experience (DMT > 0) in the entire population was 60%, and among adults caries experience was 76%. Women had significantly higher caries experience than men (*p* < 0.05). Caries was most prevalent in the molar teeth and least prevalent in the incisors and canines. Significant age-related increases in tooth wear were found, and a positive correlation between wear in molars and incisors (*p* < 0.001). Other clinical findings were signs of apical lesions, crowding of teeth, aplasia, non-erupted canines and calculus.

**Conclusions:**

Findings show that dental pathological lesions affected a majority of the studied population, and indicate that women were more predisposed to dental disease than their male counterparts. Results are discussed from a multi-factorial explanation model including dietary, physiological and cultural etiological factors.

## Background

In archaeology, examination of human jaws and teeth has long been used to study biological, physiological, and cultural aspects of societies from which studied individuals originate. Pathological and physiological dental lesions in human remains provide valuable information regarding diet, food preparation and nutrition [1–4], transitional changes within societies [5–7] and further reflects some most relatable human life qualities such as external appearance, pain sensation and masticatory function.

The variation in prevalence of dental diseases, for example in relation to age and sex, can assist in the identification of nutritional and biocultural differences in intra- and interpopulation comparisons. Since dental tissues preserves well post mortem due to their low content of organic matter, and dental procedures such as restorations were rare in the past, caries epidemiology can be studied in its original shape [8].

A wide range regarding prevalence of dental caries in archeological contexts has been reported during diverse time periods, but with an increase in occurrence from ancient to modern times. In Neolithic Europe, populations display a caries frequency of 0–9% of teeth [9–12] while a recent study of a Swedish Viking Age population revealed a caries prevalence of 12% of teeth studied [13]. Studies of dental caries in British populations dating from the Iron Age to modern time indicate that the caries disease, in terms of prevalence, distribution and affected sites of the teeth, has undergone little change during the 200-year interval from the Iron Age to late mediaeval times. From the seventeenth century, changes which indicate a shift towards increasing frequency have been recorded. Moreover, the sites predisposed to caries appear to have changed during the same time period from a high incidence of root caries to a higher number of lesions at the contact surface of the crown, as well as occlusal caries [14–16]. According to the literature 40–60% of the mediaeval and early modern day Scandinavians had experienced caries, where 5–15% of the teeth had at least one carious lesion [17–22].

Studies of prehistoric and historic populations indicate that prevalence of caries is subordinated to the survival of an individual, and that the disease has been progressive during the human life span, thus correlating prevalence with age [4, 21, 23, 24]. In modern times, however, this correlation is not as strong, due mainly to dietary changes, access to dental care and the use of fluoride [25].

Formation of caries lesions is the result of complex time-dependent interactions between acid-producing bacteria, fermentable carbohydrates and host factors including tooth and saliva. The strong relationship between dental caries and diet is well established [26–28], and the cause of caries has historically been the same as it is today, namely the result of dietary carbohydrate fermentation by endogenous bacteria (e.g. Streptococcus mutans) in the oral cavity into acids [29]. Although sucrose is known to be the main cariogenic component in our modern diet [26], starch may also contribute to caries development [30, 31]. However, the disease is of multifactorial origin, with contributing factors such as anatomy of the tooth itself by facilitation of bacterial film retention in pits and fissures, physiology including mechanical oral clearance and gland function, and salivary factors such as quality and amounts of saliva. Also, genetical and socioeconomical factors are today known to contribute to the disease [32, 33].

Sex-related differences regarding caries prevalence has been studied in several populations, of which some show differences [7, 34–37] while others fail to demonstrate such relationship [24, 38–40]. Sex differences regarding dental caries has traditionally been explained by diverse dietary patterns amongst men and women, and related to sociocultural aspects of food preparation, and its supply and dispersion. Lately hypothesis has emerged based on reproductive ecology, where gender differences in caries frequency may be attributed to age- and fertility related shifts in oral biology. The theory emphasizes the female hormones including estrogen as risk factors related to higher susceptiveness to caries, especially in pregnant women [7, 41].

Tooth wear is generally strongly associated with age, related to an overall greater exposure to abrasive elements over time [4]. Potential aetiological factors for dental wear include the function and parafunction of the jaws, diet, diseases, saliva and bite force [42], and environmental factors such as occupation may affect the development and progression of dental wear [43]. Impaired tooth quality could also be related to poor nutrition as well as diseases and lead to a greater degree of tooth wear [44]. Extensive tooth wear may lead to pulpal inflammation and even abscesses [44]. Most studies suggest that tooth wear rates have decreased from prehistoric to modern times [45].

During recent archaeological excavations in Gothenburg in south-west Sweden, human skeletal remains have been collected dating from around year 1500–1620. The remains provide a valuable source of knowledge regarding living conditions in early modern towns, as well as a measurement of health status among the inhabitants. Especially so since the town only existed for around 200 years, after which the site was abandoned and the town and all inhabitants repositioned to a new geographical location. This provides a rare opportunity to study an archeological time capsule from an early modern age Swedish settlement in its original form. Archaeologists have researched the site and publications are describing social, historical and geographical aspects, but no publications on dental health have yet been published [46, 47].

### Aim

The aim of this study was to evaluate the occurrence of dental pathological lesions, primarily dental caries and tooth wear, in an early modern population from Gothenburg, Sweden (New Lödöse) and analyze the results in a bioarcheological context with special reference to sex differences.

## Methods

### Materials

The study population was obtained from Västergötlands museum, and comprised a random selection of the skeletal remains of 308 individuals from a total population of 700 individuals. The graves date from around 1500–1620 and were discovered during the excavation of the city of New Lödöse (Nya Lödöse). New Lödöse was situated in the area of the present city of Gothenburg from 1473 to 1624 [48]. It was a small city with at most 1,200–1,300 inhabitants and was a trading center for a variety of goods. The churchyard in New Lödöse was in use in 1500–1620. The graves are placed tightly together and, in many cases, they are also placed in layers on top of one another. The human skeletons are generally very well preserved thanks to the waterlogged humid soil. The sex distribution of the buried individuals reveals a majority of men (60%) among the adult town dwellers. Child mortality in New Lödöse was high, as almost half the excavated graves contained the remains of children. Only one third of newborn infants reached adulthood. The rest of them died at a young age, most often during their first year of life, due to infectious diseases. A total of 80% of the examined individuals were found to be adults and 20% adolescents and children. Most of the adults died between the ages of 20–40 years. Despite the relatively short life span, their bodies were visibly affected by hard work. During osteological examinations, more or less advanced skeletal changes were found in the spines of most individuals and many of them also revealed traces of severe degenerative joint diseases.

The examined 308 individuals were the first remains found in the excavation of the graveyard that met the inclusion criteria, and at the point of odontological examination approximately 350 graves had been excavated. Later archeologists continued the excavations, resulting in around 350 additional remains which have not been possible to examine in this work. Inclusion criteria was persisting tooth or jaw (in whole or fragmented) with or without alveoli (mandible or maxillae). Greatly damaged jaws were excluded. An osteologist (MV) determined age and sex using standard techniques described by Buikstra & Ubelaker [49]. Age distribution in the population studied was 0–50 + years, with the majority in the age range of 30–40 years. The population was divided into two major groupings, one adult group and one child group. This distinction was made in accordance with permanent or deciduous/mixed dentition. Adults with only solitary deciduous teeth remaining and at age > 16 years were placed in the adult group.

## Methods

The dentitions were assembled and teeth and alveolar bone were cleaned with soft brushes before examination. Examinations included visual inspection and the use of a dental probe under a strong light source. Photographs were taken for illustrative purposes. Number of teeth, tooth wear, carious lesions, apical pathology and any deviating findings were recorded by two dentists (CB and LN), during a time period of 12 months.

To determine whether a tooth was lost post or ante mortem, the alveolar bone was examined. When the alveolar socket was empty, without signs of healing, the tooth was determined to have been lost post mortem. A third molar was only recorded as lost ante mortem if clear signs of its existence were found. In the event of root remains, these were recorded as remaining teeth. All retained roots were registered as remaining teeth. If the crown of a tooth was fractured post mortem, this tooth was recorded as lost post mortem.

### Carious lesions

Only cavitated lesions in the crown or/and root of a tooth were registered as carious lesions. Colour changes in the enamel were not registered as caries. The lesions were classified by their overall location in the dentition, location by site and number of surfaces involved. The location of the lesion was recorded and classified into one of the following categories: 1) occlusal, 2) mesial, 3) distal, 4) lingual/palatal and/or 5) buccal, in cases of crown caries, and 6) root, if located on the root surface. A tooth was considered to be carious if it had at least one carious lesion. When a lesion extended into more than one surface, all the affected surfaces were noted. Where only a carious root fragment was observed, all surfaces were recorded as affected. Caries experience expressed as DMT (number of Decayed and Missing Teeth) was calculated for each individual. When calculating DMT, missing teeth were only recorded when a tooth was lost ante mortem. An individual was regarded as caries free if DMT = 0. The number of individuals with detectable carious lesions was also calculated, in addition to the DMT index.

### Tooth wear

Severity of dental wear was recorded through visual inspection using the scale created by Johansson et al. [50] from 0 to 4: 0 = no visible facets in the enamel, 1 = marked wear facets in the enamel, 2 = wear into the dentine, 3 = extensive wear into the dentine and 4 = wear into the secondary dentine. The scale was used to register attrition on the first incisors and first molars in the lower jaw. If the first incisor/molar was present in both quadrants, the registration was performed on the one with the greatest degree of tooth wear. In cases where these teeth were missing, no registration was made. Registration was only performed on permanent teeth. Thus, if only primary teeth were present, no registration was carried out.

### Other oral conditions

Other oral conditions found by inspection and the use of a dental probe were noted, in addition to those mentioned above. This included apical pathology visible in the bone by inspection, anatomical variations and other deviating findings.

### Statistical method

For the statistical analysis, the SPSS program was used. Using this program, caries experience, expressed as the number of Decayed Missing Teeth (DMT), and tooth wear were compared in relation to sex and age. Both parametric and non-parametric methods were employed for the statistical analysis of the material. The non-parametric chi-square test was used to analyse caries and tooth wear in adult men compared with women. No gender differences were analysed regarding children due to the high number of individuals with unknown sex. Spearman’s rank correlation test was used to examine the age-caries correlation and age-tooth wear correlation. Statistical number of prevalence was set at *p* < 0.05. Plots and column diagrams were constructed using Microsoft Excel software.

The inter-examiner reliability was tested repeatedly during the analysis, but without any calculation of Kappa values. At the beginning of each day of examinations, an examiner calibration was performed by the two examiners on the first randomly selected skull. In the event of uncertainties during the following examinations, the other examiner was consulted. In the event of uncertainty about whether or not caries was present, no caries was registered.

## Results

The study material comprised dentitions from 205 adults and 103 children. Among the adults, the sex distribution was 119 males (58%), 81 females (40%) and five of unknown sex (2%). Among the children, the sex distribution was one female, one male and 101 individuals of unknown sex. The mean age at death for adult men and women in the studied population was 33 years for both groups, and the median was also the same for men and women namely 32.5 years. A total of 4,951 teeth were examined in adults and 1,660 teeth in children, making a total of 6,611 teeth examined. These findings are presented in Table 1.

**Table 1:** Number of examined and carious teeth in the children and adults

	Number of teeth	Carious teeth (%)
Child	1660	81(5%)
Adult	4951	636 (13%)
Total	6611	71 (11%)

### Tooth loss

**Fig. 1:**
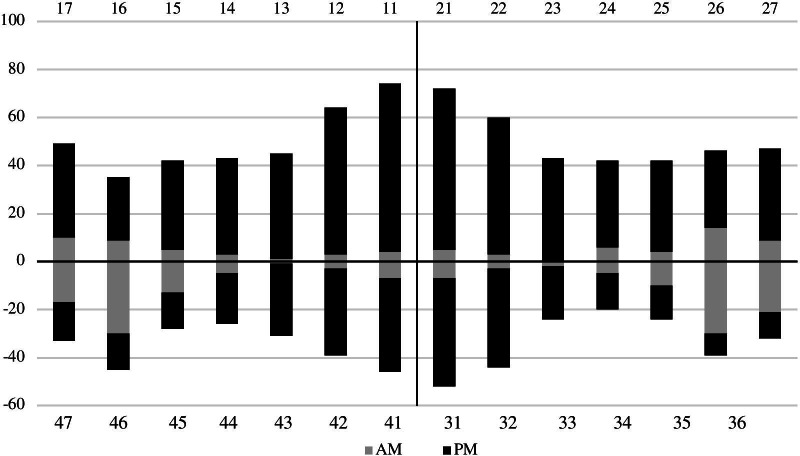
Number of teeth lost ante mortem (AM) and post mortem (PM) by tooth number

The total number of lost teeth in the adult population was 1,609, of which 82% were lost post mortem and 2% ante mortem. The total number of lost teeth also included non-erupted third molars; this category consisted of 132 teeth. Thus, 16% of the third molars had not erupted. The teeth most commonly lost post mortem were upper and lower incisors, followed by upper molars. The teeth most commonly lost ante mortem were first lower molars (Fig. 1).

### Caries

#### Caries prevalence and experience (DMT) on an individual basis

The caries prevalence and caries experience are presented in Table 2. A total of 55% of the individuals had at least one carious lesion: 68% of the adults and 28% of the children. Among the adult individuals with no carious lesions, 71% were men and 26% were women. The caries experience in the population, expressed as DMT > 0, was 60%. Caries experience was found in 76% of adults and 28% of children. Among adults, 29% of men and 16% of women were lacking caries experience (DMT = 0) (Table 2). A statistically significantly higher caries experience was found in women compared with men, comparing DMT = 0 and DMT > 0 (*p* < 0.05). No significant difference in the number of carious lesions was found when comparing men and women. When evaluating the correlation between age and caries prevalence for all individuals, no significant correlation was found (ns).

**Table 2:** Number of individuals with at least one caries lesion registered, caries free individuals, and individuals with or without decayed and missing teeth (DMT) according to sex in the adult population, and findings from statistical analysis by comparison of men and women

	Male	Female	Unknown	*p* value
Caries registered	73	64	3	0.52
No caries registered	46	17	2	
DMT > 0*	85	68	3	0.041**
DMT = 0	34	13	2	

^*^ Individuals with carious teeth and/or teeth lost ante mortem

^**^ Significant difference

#### Caries prevalence by tooth and surface

**Fig. 2:**
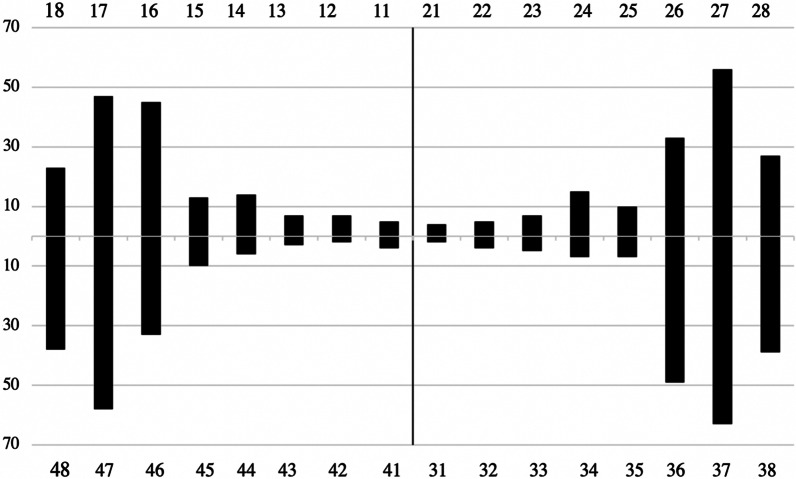
Number of teeth with caries by tooth number in the adult group

The caries prevalence by tooth and surface is presented in Table 3 and Fig. 2. In the studied material, 11% of the teeth showed at least one carious lesion. The number of carious teeth per individual varied between 0 and 20. Among children, 5% of the teeth showed at least one carious lesion, while among adults 13% of the teeth showed at least one carious lesion. Figure 2 shows the number of teeth with caries among adults. In the adult group, 568 (11%) teeth had crown caries and 103 (2%) had root caries. A smaller proportion of the teeth (0.1%) had cavities in both the crown and root. The occlusal area was the surface most susceptible to caries, followed by the approximal surfaces. The teeth where caries was most commonly found were the second molars.

**Table 3:** Carious lesions in adults by surface affected

Surface	Occlusal	Mesial	Buccal	Lingual	Distal	Root	Total
No of surfaces affected	492	172	161	132	184	116	1257
Percentage of surfaces affected	39%	14%	13%	11%	15%	9%	100%

Missing and carious teeth in adults are presented according to the type of tooth (Table 4). A total of 30% of the molars were lost in the adult population and the number of molars with at least one carious lesion was also 30%. Of the premolars in the adult population teeth, 16% were lost and 6% had at least one carious lesion. Among incisors and canines in the adult population, 24% were lost and only 2% had at least one carious lesion.

**Table 4:** Missing and carious teeth in relation to tooth group in the adult population

Tooth category	Expected number of teeth	Number of missing teeth	Teeth lost post mortem	Teeth lost ante mortem	Number of examined teeth	Carious teeth (% of examined teeth)
Incisors and canines	2460	602 (24%)	562 (23%)	40 (2%)	1858	41 (2%)
Premolars	1640	269 (16%)	215 (13%)	54 (3%)	1371	83 (6%)
Molars	2460	738 (30%)	536 (22%)	202 (8%)	1722	512 (30%)
Total	6560	1609	1313	296	4951	636

### Tooth wear

The degree of attrition on incisors and molars in adults and children is shown in Table 5*.* In adults, the molars displayed significantly more advanced wear than the incisors (*p* = 0.032). In children, the incisors showed numerically more advanced wear compared with the molars (ns). A positive correlation between age and degree of attrition was found for both incisors and molars, with a stronger correlation for molars than for incisors (r = 0.541, *p* < 0.01 and r = 0.698, *p* < 0.01 respectively). A positive correlation was also found when comparing the degree of attrition on incisors and molars (r = 0.627, *p* < 0.01). No significant differences were found in relation to sex (ns).

**Table 5:** Degree of tooth wear of incisors and molars in adults and children

	Incisors		Molars	
Degree of dental wear	Adults	Children	Adults	Children
0	0	8	2	26
1	16	26	24	20
2	93	11	54	2
3	45	1	67	0
4	26	0	43	1
Unknown	25	57	15	54

### Other findings

Additional findings of different oral conditions recorded are presented in Table 6, including unusual tooth wear, such as a so-called pipe-mark on a permanent molar, and abrasion due to some kind of work. Many teeth showed clinically detectable apical lesions in the alveolar bone. The individual with most teeth affected had seven teeth with clinically detectable apical infections. Other findings included the crowding of anterior teeth, in both the maxilla (in one individual) and the mandible (in five individuals). Seven cases of palatal eruption or retention of the canine teeth were found among adults. One child had lingual and palatal eruption of the permanent canines with persisting deciduous canines in both the maxilla and mandible. Persisting deciduous teeth were found in three adults and peg-shaped teeth were found in three other adults. Two of these individuals displayed a peg-shaped lateral, while the other individual had a peg-shaped premolar.

**Table 6:** Other oral conditions identified in adults and children. Number of children with findings in parentheses

	Number of individuals	
Type of finding	Adults	Children
Apical lesion	43	0
Palatal/lingual eruption/ retention of canine teeth	7	2
Persisting deciduous teeth	4	1
Missing permanent tooth and no persisting deciduous tooth	2	0
Root resorption	2	0
Crowding of teeth	6	0
Peg-shaped tooth	3	0
Enamel mineralisation disturbances	3	3
Abnormal tooth wear	2	

**Fig. 3:**
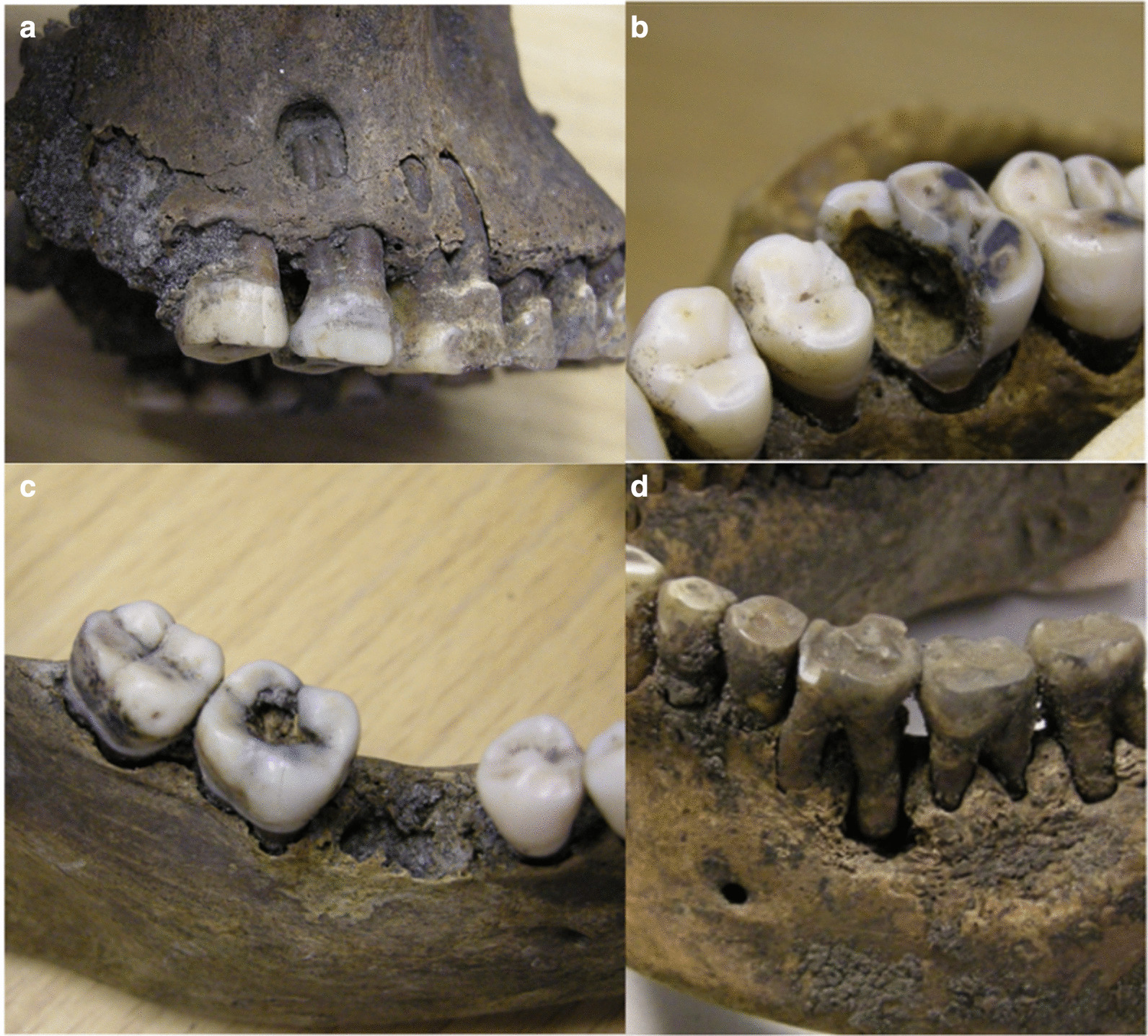
**a** Tooth 17 with a distal caries lesion and apical infection perforating the buccal bone. **b** Mesial and occlusal cavity in tooth 26. **c** Occlusal cavity in tooth 47, and ante-mortem loss of tooth 46. **d** General attrition and apical infection in the distal root of tooth 36

Root resorption was found clinically in two cases; the first case involved resorption of both maxillary central incisors, and in the other case resorption of a maxillary second molar. In three individuals from the adult group and three individuals from the child group, enamel mineralisation disturbances were noted. All these were found in the permanent dentition and all except one were found as general disturbances, which were found locally in one tooth. In addition to these findings, many individuals showed a major accumulation of calculus. For illustrative purposes, see attached photos in Fig. 3.

## Discussion

Prevalence of teeth lost ante mortem was 4% in the adult group. By analyzing the alveolar bone, it is possible to determine whether a tooth was lost during life and not post-mortem, but it is not possible to determine the reason for its loss with any certainty [51]. Nor is it possible to decide whether a tooth lost ante mortem has been exfoliated, or extracted by man. Even though caries is regarded as the major cause of tooth loss [52], periodontitis, gross attrition and trauma are believed to have been important causes of tooth loss in prehistoric times [53, 54]. Moreover, symbolic tooth extractions may have taken place in some cultures [51], and the surgical procedure of extracting teeth is an ancient practice that is known to have been used in many populations to treat tooth pain [24]. Ante-mortem tooth loss is sometimes used as an indicator of oral health, even though the aetiology of missing teeth can be of non-pathological origin. In historical populations, comparing teeth lost ante and post mortem is significant when investigating caries prevalence and dental health [17], due to the fact that teeth lost both ante- and post-mortem represent an uncertainty when evaluating caries prevalence.

In the present material, 20% of studied teeth were found to have been lost post mortem in the adult population. It is impossible to know how much caries and tooth wear these teeth experienced, which may affect the accuracy of the total caries prevalence and should be taken into consideration. However, in adults, approximately half the teeth lost post mortem were incisors and canines which, in this and other studies, have been shown to be least affected by caries [55, 56]. Tooth loss post mortem was more common in the maxillae compared to the mandible, which is in accordance with previous studies [20, 34]. This is explained by the bone volume differences in the jaws where the maxilla has a more porous bone structure and less bone mass than the denser mandible.

The teeth most commonly lost ante mortem were 36 and 46, and since mandibular first molars were the teeth with highest caries prevalence, it is possible to conclude that 36 and 46 were lost to the largest extent due to excessive caries. Undoubtedly, the causes of tooth loss in studied population can also be related to periodontal, infectious and attritional pathology. However, it is not possible to determine whether they were extracted by man or exfoliated. To fully understand the causes by which teeth were lost in this population, a comprehensive examination of periodontal disease would be of value.

Regarding post-mortem, the teeth most commonly lost were the upper incisors and upper third molars, probably due to the conical root anatomy of the incisors antagonising retention in the jaws post mortem, and the unfavorable anatomy of the third upper molars, as well as post mortem fractures of the tuber maxillae.

Present study found that 55% of the individuals had at least one carious lesion, and 13% of teeth had cavities. Other historic populations have shown a similar caries prevalence. In literature, the caries prevalence of European populations fluctuates during the Middle Ages and early modern times, but it affects up to 50% of the individuals in the early period [23, 24, 34, 57] and increases in later mediaeval times and early modern-day populations [58, 59]. In Scandinavian populations, the caries prevalence during the Middle Ages has been reported to be around 40–60% [18, 19, 21, 22, 31, 60]. Thus, the findings in this paper are in line with previous findings where one study closely matches the time period and area of excavation (Gothenburg, Sweden) and also counterparts the present caries prevalence found [20].

Several studies, of both modern and prehistoric populations, have revealed sex related differences regarding caries prevalence. Many have found a higher caries prevalence in women compared to men [17, 21, 24, 51] but some work has stated the opposite relation [35], while others failed to find such relation. Where disparity has been found, it is often explained by dietary differences [61], hormonal influences on the local oral environment [41] and/or sex differences regarding dental treatments [24]. It is well-known that in many societies during the Middle Ages, women were responsible for food preparation and cooking, thus probably exhibiting a more cariogenic eating pattern. Statistical difference was in current work found for caries experience (DMT) between men and women, with a significantly higher caries experience in women. A numerical, but not significant, difference was found regarding individuals with one or more cavity when comparing men and women, where women were more predisposed (Table 2). These findings indicate that women suffered poorer dental health than their male counterparts, but apparently does not give explanation to why. It is of most importance to consider the complexity of the disease when drawing explanatory models regarding dissimilarities in caries experience, since it is well-known that many intrinsic factors play large role regarding caries susceptibility. It would be naïve to simply lay the explanation on a single factor. The causes of current findings (the higher caries experience among females) are believed to lie in a multi-factorial model of explanation, due to the nature of the disease, including dietary patterns, salivary components, cultural- and social constructs and perhaps genetic and hormonal influences. However, the true value of this work should be the deeper understanding that data provides about the historical population studied. New Lödöse was as previously mentioned a new community, populated with a broad spectrum of inhabitants from diverse rural and urban areas in the region. Additionally, several nationalities, with perhaps slightly different cultural background, gathered in this fast-growing new town. Tax records reveal that many different occupations were represented in this heterogeneous society, such as merchants, shoemakers, tailors, farmers, carpenters, smiths, weaver, barbers, etcetera. Nearly all individuals mentioned in the tax records were men, even if women and children were just as involved in the family business as their husbands, brother or fathers. The women exceptionally listed are widows continuing the work of their late husbands. Evidence from the town cemetery discloses a surplus of men among the buried [62], reflecting that New Lödöse also by numeral was a male society. The situation for women in New Lödöse in the sixteenth century was probably harsh in many ways with considerably less power, less money, less freedom and higher vulnerability in society compared to men. In the sixteenth century the need to control women, their life and everyday social intercourse was immense. Moving into town, inhabitants were no longer subject to the strong social control of the small rural communities were most of them probably originated, meaning new opportunities but also danger. Historical sources from New Lödöse give witness of numerous conflicts and court trials where inhabitants accuse one another for loosely living, rule breaking sexual behaviour, infidelity, inter-personal violence and theft. Even if the records reveal that men were more exposed to violence in daily life compared to women, there is evidence of physical abuse of females ranging from beating to rape [63]. Assessment of age at death in New Lödöse presents clear evidence that a higher proportion of women compared to men died before the age of 40, reflecting the general trend in medieval and early modern historical populations. Higher mortality of women in fertile age is most often explained by stress and complications during pregnancy and child birth which is probably true, but explanation might also need to include other factors. Excess female mortality of young adults could indicate that early modern women due to conditions during childhood suffered poorer health than men, consequently being less equipped to face diseases and other physical stresses. Girls possessed lower status in society compared to boys and may have been of less priority if there was shortage of food. If so, the poorer dental status of women noted in the skeletal material from New Lödöse and other places, might partly be a result of unequal living conditions for girls during childhood. Conclusively, not only did females during this time have impaired possibility to autonomy, lived shorter lives and bare the physical burden of childbirth and housekeeping, but they must also have suffered in a larger extent because of their poorer dental status compared to males.

In spite of a diet with a presumably low sugar content, caries affected a large number of individuals living in New Lödöse. There is evidence that caries in early modern humans was not due to refined carbohydrates but rather to starchy plant foods [64]. It is known that New Lödöse was a trading center for a variety of goods, and trading ships sailed mainly to the North Sea region, including Holland, England and northern France. Wood, beer, wine, grains and fish, along with spices such as cloves, ginger root, pepper and cumin, are known to have been traded, as well as honey and candi-sugar. It is not completely established whether any changes occurred in the Scandinavian diet during the late Middle Ages compared with earlier periods. It is, however, known that sugar was imported by Great Britain from the beginning of the fifteenth century, initially in small quantities, while sugar was probably not introduced to the Scandinavian countries until later [14]. It is also known that the consumption of sucrose increased in Europe during the seventeenth century due to the establishment of the West Indian sugar industry [16]. Furthermore, some of the individuals buried in New Lödöse might not have lived permanently in the region and may even have been international visitors. The sex distribution of the buried individuals with a majority of men (60%) among the adult town dwellers may be a result of labor migration due to the type of employment that was offered in the town. It has been suggested that an increase in the intake of grain and food products from market gardening may have contributed to the development of caries during the time period. Moreover, the intake of starches might have contributed to the development of the caries disease [31].

The high prevalence of caries in the studied material could be explained by a higher intake of refined carbohydrates and sugars during early modern times in comparison with the early Middle Ages. Another factor to consider in relation to the varying caries prevalence found in different historical populations is fluctuating fluoride levels in drinking water. No information is available regarding the fluoride content of the water in New Lödöse during early modern times. Present data show that children had less caries experience than adults, which corresponds well with the results of previous studies [15, 21, 23, 24]. This could be explained by the slow rate of caries progression, thus taking long time for carious lesions to develop. The type of foods consumed during the sixteenth and seventeenth century, including carbohydrates that were less processed and thus less readily available for bacteriological fermentation provides an explanation for this, however, there might be additional factors affecting progression rates of caries disease during historical times, such as microflora, dental hygiene, and salivary components.

In the studied dentitions, a large difference was found between caries prevalence in molars (30%), premolars (6%) and incisors and canines (2%). This is in line with previous studies of prehistoric skull material [21, 23, 24] and might be explained by the more intricate fissure systems in molars than in other tooth groups thus the more frequent accumulation of bacteria as well as their use during mastication. This is supported by the findings in that 39% of the carious lesions in this material were situated on the occlusal surface.

The study population showed a greater amount of tooth wear compared with modern populations [44], and findings revealed a strong correlation between increasing age and degree of tooth wear. A correlation between the degree of tooth wear in molars and incisors were found, suggesting that individuals with a high degree of wear on molars also had increased wear on their incisors. All these findings are in line with previous findings in archaeological material [44]. Iron Age and early mediaeval dentitions from Skara, Sweden, as well as early modern-day dentitions from Gothenburg [65, 66] displayed similar tooth wear patterns (more severe on molars than incisors) as the adults examined in this work.

A high prevalence of apical lesions was found, even without using radiographic imaging. Using radiographic imaging, more infections would probably be detected. A broad pit or fistula around the root apex is a clear sign of an apical lesion [67], however, the first signs of an apical infection can only be observed radiographically, in the loss of the continuity of the lamina dura [35]. The high prevalence of infections indicates that it was not uncommon for the inhabitants of New Lödöse to suffer pain due to dental disease, and it must be considered that the dental pathologies severely affected the well-being of these early modern age individuals.

The complexity of dental health includes in addition to caries, tooth wear and apical infections, factors such as periodontal disease, anatomical and masticatory variations. Enamel hypoplasia, crowding of teeth and abnormal eruption were all found in this population, but was not the focus of current work. In addition to this, persisting deciduous teeth, indicating aplasia or retained teeth, root resorption of teeth and peg-shaped teeth were found. By expanding examinations to include radiographic imaging, these phenomena could be examined further. Since emphasis of current work was to study dental caries and tooth wear, it would be of interest to complement this work with a complete and in-depth examination including analysis of periodontal disease, other dental pathology and anatomical variations in the studied population.

## Conclusions

The study of dental pathologies, and especially caries, is an important field in bioarcheology because of the extensive understanding of populations that could be gained from these findings. In the studied population, dental caries affected the majority of the inhabitants, despite a diet presumably low in sugars. There was a significant sex difference regarding dental health which favored males. The susceptibility of caries amongst females should be put in the complex physiological, cultural and biological context which mediates and effects the caries disease. A majority of the population suffered from poor oral health, with a high prevalence of caries, tooth wear, and root infections, and pathology augmented with increasing age. This in a time when assuagement from the dental discomforts assumingly was rare, as was dental procedures beyond extractions. Discomfort due to toothache, food impaction, masticatory problems and perhaps even esthetical considerations was probably common in the studied population and provides a sensibility in the understanding of these unique historical remains.

## Data Availability

The datasets used and/or analysed during the current study are available from the corresponding author on reasonable request.

## Abbreviations

DMT: Decayed Missing Teeth
SPSS: Statistical Package for the Social Science
